# TLR9 and STING agonists cooperatively boost the immune response to SARS-CoV-2 RBD vaccine through an increased germinal center B cell response and reshaped T helper responses

**DOI:** 10.7150/ijbs.81210

**Published:** 2023-05-29

**Authors:** Jing-Xing Yang, Jen-Chih Tseng, Chih-Feng Tien, Chia-Yin Lee, Yi-Ling Liu, Jhe-Jhih Lin, Pei-Ju Tsai, Hung-Chun Liao, Shih-Jen Liu, Yu-Wen Su, Li-Chung Hsu, Jen-Kun Chen, Ming-Hsi Huang, Guann-Yi Yu, Tsung-Hsien Chuang

**Affiliations:** 1 Immunology Research Center, National Health Research Institutes, Miaoli, Taiwan.; 2 National Institute of Infectious Diseases and Vaccinology, National Health Research Institutes, Miaoli, Taiwan.; 3 Institute of Molecular Medicine, College of Medicine, National Taiwan University, Taipei, Taiwan.; 4 Graduate Institute of Immunology, College of Medicine, National Taiwan University, Taipei, Taiwan.; 5 Institute of Biomedical Engineering and Nanomedicine, National Health Research Institutes, Miaoli, Taiwan.; 6 Department of Life Sciences, National Central University, Taoyuan, Taiwan.; 7 Program in Environmental and Occupational Medicine, Kaohsiung Medical University, Kaohsiung, Taiwan.

**Keywords:** Adjuvant, CpG-oligodeoxynucleotide, Cyclic dinucleotide, SARS-CoV-2, Stimulator of interferon genes, Toll-like receptor, Vaccine.

## Abstract

Vaccines are a powerful medical intervention for preventing epidemic diseases. Efficient inactivated or protein vaccines typically rely on an effective adjuvant to elicit an immune response and boost vaccine activity. In this study, we investigated the adjuvant activities of combinations of Toll-like receptor 9 (TLR9) and stimulator of interferon genes (STING) agonists in a SARS-CoV-2 receptor binding domain protein vaccine. Adjuvants formulated with a TLR9 agonist, CpG-2722, with various cyclic dinucleotides (CDNs) that are STING agonists increased germinal center B cell response and elicited humoral immune responses in immunized mice. An adjuvant containing CpG-2722 and 2'3'-c-di-AM(PS)2 effectively boosted the immune response to both intramuscularly and intranasally administrated vaccines. Vaccines adjuvanted with CpG-2722 or 2'3'-c-di-AM(PS)2 alone were capable of inducing an immune response, but a cooperative adjuvant effect was observed when both were combined. CpG-2722 induced antigen-dependent T helper (Th)1 and Th17 responses, while 2'3'-c-di-AM(PS)2 induced a Th2 response. The combination of CpG-2722 and 2'3'-c-di-AM(PS)2 generated a distinct antigen-dependent Th response profile characterized by higher Th1 and Th17, but lower Th2 responses. In dendritic cells, CpG-2722 and 2'3'-c-di-AM(PS)2 showed a cooperative effect on inducing expression of molecules critical for T cell activation. CpG-2722 and 2'3'-c-di-AM(PS)2 have distinct cytokine inducing profiles in different cell populations. The combination of these two agonists enhanced the expression of cytokines for Th1 and Th17 responses and suppressed the expression of cytokines for Th2 response in these cells. Thus, the antigen-dependent Th responses observed in the animals immunized with different vaccines were shaped by the antigen-independent cytokine-inducing profiles of their adjuvant. The expanded targeting cell populations, the increased germinal center B cell response, and reshaped T helper responses are the molecular bases for the cooperative adjuvant effect of the combination of TLR9 and STING agonists.

## Introduction

Infectious diseases are a major threat to human life and can lead to global health crises. Their serious social impact is clear from the ongoing COVID-19 pandemic caused by the severe acute respiratory syndrome coronavirus 2 (SARS-CoV-2), which began in late 2019. According to the World Health Organization, as of July 2022, SARS-CoV-2 has infected 551 million people and resulted in 6.3 million deaths worldwide. SARS-CoV-2 primarily spreads among people through aerosols and respiratory droplets produced from coughs, sneezes, or exhaling 1-3. After inhalation into the respiratory tract, the virus utilizes its spike (S) protein to interact with the cellular receptor angiotensin-converting enzyme 2 (ACE2) to enter cells. S proteins are located on the viral envelope. They form homotrimers and are processed into S1 and S2 subunits by furin protease. The S1 subunit contains a receptor binding domain (RBD) that interacts with ACE2 during infection. Following binding, the S2 subunit is cleaved by TMPRSS2 protease to trigger virus-host membrane fusion 4-6.

Vaccines are powerful tools to control viral spreading and epidemic diseases. To date, 40 vaccines have been approved for use or received emergency use authorization (EUA) for COVID-19 in at least one country 7. Most are inactivated viruses, protein subunits, non-replicating viral vectors, and mRNA vaccines. Viral vector and mRNA COVID-19 vaccines are the first of their kind to be approved for use in humans. They are manufactured from the latest vaccine technologies but still have limitations. For example, both types of vaccines are associated with adverse effects including myocarditis, thrombosis, and anaphylaxis. Additionally, ultra-cold supply chain equipment is required for mRNA vaccines, usually not feasible in most developing countries 8-10. Protein subunit vaccines mainly use recombinant proteins as antigens. Current COVID-19 vaccine designs use recombinant S protein or the RBD domain as the target antigen to induce neutralizing antibodies to block SARS-CoV-2 entry. Nevertheless, recombinant antigens typically show weak immunogenicity even in the presence of aluminum salts, which have been used as an adjuvant for the last century. Thus, effective adjuvants for vaccines still require exploration 11-13.

Pattern recognition receptors (PRRs) are essential for innate immune cells to detect microbial pathogens to initiate an immune response against invading microbes. Based on their ligand recognition and structural features, PRRs fall into several major groups: Toll-like receptors (TLRs), C-type lectin receptors (CLRs), nucleotide-binding oligomerization domain-like receptors (NLRs), retinoic acid-inducible gene I-like receptors, and cytosolic DNA sensors including absent in melanoma-2-like receptors. Activation of these PRRs induces an early phase antigen-independent innate immune response that subsequently leads to the activation of an antigen-dependent adaptive immune response 14-16. Because of this, PRRs and their signaling molecules are targets for effective adjuvant exploration. Examples include TLR9, which is one of ten human TLRs, and stimulator of interferon genes (STING), which is a signaling molecule downstream of cyclic GMP-AMP synthase (cGAS), a cytosolic DNA sensor 17-20.

TLR9 senses microbial unmethylated CpG-dideoxynucleotides containing DNA (CpG-DNA). Synthetic phosphorothioated CpG-oligodeoxynucleotides (CpG-ODNs) mimic the function of microbial CpG-DNAs in the activation of TLR9. Activation of TLR9 by CpG-ODNs induces antigen presentation and cytokine production in antigen-presenting cells. These promote the T helper 1 (Th1) polarization of CD4^+^ T cells and result in the expansion of antigen-specific CD8^+^ T cells and antibody-producing plasma B cells. Thus, CpG-ODNs have been investigated as vaccine adjuvants 21-23. A CpG-1018 has been formulated as an adjuvant in a licensed hepatitis B vaccine and two EUA-approved COVID-19 vaccines. Additionally, CpG55.2 was formulated in a vaccine EUA-approved for COVID-19 24-27. The cGAS/STING pathway detects cytosolic DNA from microbes to induce host immune responses. Upon ligand activation, cGAS triggers the formation of cyclic GMP-AMP (cGAMP) from GTP and ATP. cGAMP then binds to STING, activating signal transduction, leading to the transcription of type I interferons (IFNs) and NF-κB-controlled inflammatory genes 28, 29. Natural STING agonists include 2'3'-cGAMP, 3'3'-cGAMP, c-di-GMP, and c-di-AMP. Among these cyclic dinucleotide (CDN)-based STING agonists, 2'3'-cGAMP is produced by cGAS in mammalian cells, and the others are produced by cGAS in bacteria. Furthermore, STING is activated by synthetic CDNs and structurally unrelated small molecules, the former including thiolated 2'3'-cGAM(PS)2 and 2'3'-c-di-AM(PS)2 and the latter including DMXAA and diABZI. STING agonists are being investigated as vaccine adjuvants, but none have been used in licensed vaccines 20, 30-32.

CpG-2722 is a CpG-ODN that has a good immunostimulatory response to TLR9 from different species 33. In this study, we used the SARS-CoV-2 RBD protein as an antigen to investigate the mechanism and function of a CpG-2722 and STING agonist combined adjuvant in COVID-19 vaccines.

## Materials and methods

### Mice

Mice were purchased from BioLASCO Taiwan (Taipei, Taiwan) or the National Laboratory Animal Center (Taipei, Taiwan) and were housed at the Laboratory Animal Center of the National Health Research Institutes (NHRI). Mice used in this study were 6-8 weeks old. These mice were maintained and handled following stated guidelines. All procedures were approved by the Institutional Animal Care and Use Committee of the NHRI.

### Antigen and adjuvants

Recombinant SARS-CoV-2 RBD protein (amino acid residues Arg319-Phe541) was purchased from Elabscience (Catalog No. PKSR030521). STING ligand 2'2'-cGAMP (Catalog No. 22419) was from Cayman, and 2'3'-cGAMP (Catalog No. vac-nacga23), 2'3'-cGAM(PS)2 (Catalog No. tlrl-nacga2srs), and 2'3'-c-di-AM(PS)2 (Catalog No. vac-nacda2r) were from InvivoGen. The TLR9 agonist CpG-2722 was synthesized by Integrated DNA Technologies. The adjuvant doses used were 5 μg and 10 μg per mouse for STING ligands and CpG-2722, respectively.

### Mouse immunization

Female BALB/c mice were used for immunogenicity studies. The vaccine was administered in three doses at 10-day intervals. The mice were intramuscularly or intranasally immunized on days 0, 11, and 21 with the formulated RBD/adjuvant vaccines. Control mice received PBS. Intramuscular immunization was done through injections in the right hind leg quadriceps muscle with 50 μl of the antigen/adjuvant mix. For intranasal inoculation, 30 μl of the vaccine was dropped into the nostrils (15 μl for each nostril). Serum samples were obtained 10 days after each immunization for humoral immune response analysis. Spleens, draining lymph nodes, bronchoalveolar lavage fluid (BALF), nasal lavage fluid (NLF), and nasal tissues were collected 10 days after the final vaccination.

### Antigen-dependent immune responses and cytokine assays

Immunized mice were euthanized 10 days after the final immunization and spleens were isolated for splenocyte preparations. The cells were plated with 6 × 10^5^ cells/well in 96-well plates (Corning) and stimulated with 10 μg/ml spike RBD protein (Elabscience). After 96 h, supernatants were harvested to analyze cytokine levels. Mouse IL-4, IL-5, IL-13, IL-17, and IFN-γ were measured with commercially available ELISA kits (Invitrogen) according to the manufacturer's protocol.

### ELISA for antibody responses

Anti-S protein-specific antibody titers in serum, BALF, and NLF samples collected from immunized animals were detected with ELISA. 96-well plates were coated with 4 μg/ml recombinant S protein in PBS at 4°C overnight. Plates were washed with PBS containing 0.05% Tween-20 and blocked with 1% BSA in PBS for 1 h at room temperature. Serially diluted samples were added and incubated for 2 h at room temperature. Plates were washed and incubated for 1 h at room temperature with HRP-conjugated goat anti-mouse IgG or IgA (Invitrogen). Following washes, signals were developed with TMB substrate (Thermo Scientific) for 15 min, then the colorimetric reaction was stopped with 2 N H_2_SO_4_. The optical density was measured using a microplate reader at a 450 nm wavelength.

### hACE2-RBD competition assay

96-well plates were coated with ACE2-Fc (2.5 μg/ml, GenScript) overnight at 4°C. The plates were washed and blocked with 1% BSA at 37°C for 1 h. After washing three times, the plates were incubated with RBD-HRP (1:1000, GenScript) mixed with serially diluted serum samples at 37°C for 1 h. After washing three times, the signals were developed by incubation with TMB substrate (Thermo Scientific) at 37°C for 20 min. The reaction was stopped with 2 N H_2_SO_4_. Then, the absorbance was measured using a microplate reader at a 450 nm wavelength.

### Pseudovirus neutralization assay

A VSV-Luc-based neutralization assay was performed as previously described 34. Briefly, 1 × 10^4^ BHK21-hACE2 cells were seeded in 96-well plates. Heat-inactivated mouse serum samples (56°C for 30 min) were diluted in PRMI medium. The diluted serum samples were mixed with ~2 × 10^3^ pfu VSV∆G-FLuc/S∆19 pseudovirus at 37°C for 1 h. Then, the cells were incubated with the serum-virus mixture at 37°C for 60 min. After 24 h, the luciferase activity in cells was measured with the ONE-GloTM luciferase assay system (Promega) for infection with pseudovirus.

### SARS-CoV-2 neutralization assay

A wild-type SARS-CoV-2 neutralization assay was performed in the BSL-3 lab at NHRI. Vero cells were seeded (2.4 ×10^4^ cells/well) in 96-well plates for 24 h. Heat-inactivated serum (56°C for 30 min) from mice was serially diluted in M199 medium in 2-fold dilutions from 1:20. The diluted serum was mixed with SARS-CoV-2 virus with 200 TCID_50_ for 2 h at 37°C. The mixture was added in quadruplicate to the cells, and the cytopathic effect of each well was recorded after 4-5 days of incubation. The neutralization titer of the serum sample was calculated as the reciprocal of the highest serum dilution that prevented viral infection by 50%.

### Cell preparation and culture

Mouse splenocytes and PBMCs were isolated from C57BL/6J mice and cultured in RPMI 1640 medium supplemented with 10% FBS, 2 mM L-glutamine, 10 mM HEPES, and 1% antibiotic-antimycotic at 37°C in 5% CO_2_. To prepare the splenocytes, spleens were collected and mashed through a 70-μm nylon cell strainer (Biologix) and centrifuged at 1200 rpm for 10 min. The cells were incubated with ACK lysis buffer for 5 min at room temperature to lyse the erythrocytes, and this reaction was terminated by adding 5 ml PBS. The splenocytes were washed with PBS and plated at 2.5 × 10^6^ cells/well in a 12-well plate. Mouse PBMCs were isolated by Ficoll gradient centrifugation following the standard procedure (Ficoll-Paque Premium 1.084, GE Healthcare). Briefly, mouse whole blood was collected in heparinized tubes (BD Biosciences) and diluted with up to 4 ml PBS. The diluted blood was carefully layered on Ficoll-Paque Premium (3 ml). The samples were centrifuged at 400 × *g* at 20°C for 40 min. The PBMC-containing interphase layer was removed, washed twice with PBS, and subsequently plated at 1 × 10^6^ cells/well in a 12-well plate. Mouse embryonic fibroblasts (MEFs) were prepared from C57BL/6J mice. The cells were seeded at 4 × 10^5^ cells/well in a 12-well plate in DMEM medium supplemented with 10% FBS, 2 mM L-glutamine, 1 mM sodium pyruvate, 10 mM HEPES, and 1% antibiotic-antimycotic at 37°C in 5% CO_2_. Bone marrow derived macrophages (BMDCs) were prepared from C57BL/6J mice. Bone marrow cells were flushed from the mouse femur and tibia with PBS followed by red blood cell lysis and PBS wash, and then grown in culture medium (RPMI 1640 supplemented 10% FBS, 2 mM L-glutamine, 1% antibiotic-antimycotic, 1% MEM nonessential amino acid solution, 1 mM sodium pyruvate, 10 mM HEPES) containing 100 ng/mL FMS-like tyrosine kinase 3 ligand (Flt3-L) (PeproTech) and 5 ng/mL granulocyte-macrophage colony-stimulating factor (GM-CSF) (PeproTech). At days 3 and 6, additional culture medium containing 100 ng/mL Flt3-L and 5 ng/mL GM-CSF was added to the plate. At day 9, the non-adherent cells were collected and seeded at 2 × 10^6^ cells/well of a noncoated six-well plate.

### Flow cytometry

Spleens and draining lymph nodes were collected from the vaccinated mice, and single-cell suspensions were prepared, washed with FACS buffer (PBS containing 2% FBS), and maintained in the dark at 4°C. The viable cells were stained with Fixable Viability Stain 620 (BD Biosciences) and were then incubated with anti-CD16/32 (S17011E, BioLegend) for 10 min to reduce nonspecific antibody binding. After Fc blocking, the cell suspensions were stained for 30 min with antibodies to B and T cell surface markers: FITC-GL7 (GL7, BioLegend), PE-CD38 (90, eBioscience), APC-Cy7-B220 (RA3-6B2, BioLegend), BV421-CD95 (Jo2, BD Biosciences), BV510-CD19 (1D3, BD Biosciences), BB515-ICOS (7E.17G9, BD Biosciences), PE-CXCR5 (SPRCL5, eBioscience), BV421-CD4 (GK1.5, BioLegend), and BV510-PD-1 (J43, BD Biosciences). For intracellular staining, T cells were fixed with the Transcription Factor Buffer Set according to the manufacturer's instructions (BD Biosciences). The cells were then intracellularly stained with Alexa Fluor 647-Bcl-6 (IG191E/A8, BioLegend) for 50 min. For BMDCs, cells were stained with PE-CD11c (N418, Invitrogen), Pacific Blue-CD40 (1C10, Invitrogen), APC-CD80 (16-10A1, Invitrogen), PE-Cy7-CD86 (GL1, Invitrogen), and APC-Cy7-CCR7 (4B12, Invitrogen) for 30 min. Fluorescence was analyzed using a FACSCanto II Flow Cytometer (BD Biosciences). Data were analyzed using FlowJo software.

### RNA isolation and reverse transcription-quantitative PCR

Total RNA was extracted from mouse BMDCs, splenocytes, PBMCs, and MEFs using the illustra RNAspin Mini Kit (GE Healthcare) according to the manufacturer's protocol. First-strand cDNA was synthesized from total RNA in the presence of random hexamers using the SuperScript IV First-Strand Synthesis System (Invitrogen). Quantitative PCR was performed with the QuantiNova SYBR Green PCR Kit (Qiagen) using Applied Biosystems ViiA 7 Real-Time PCR System with gene-specific primers (Supplementary Table 1) for gene expression analysis. All primers used were synthesized by Protech Technology (Taipei, Taiwan). Target gene expression was calculated by the comparative ΔΔ cycle threshold (C_t_) method for relative quantification after normalization to *ACTB* expression.

### Hematoxylin and eosin (H&E) staining

Mice were sacrificed 10 days after the final vaccination. The nasal tissues were harvested and fixed with 10% buffered formalin. Paraffin-embedded tissues were sectioned into 5-μm tissue slides. These tissue slides were stained with H&E by the Pathology Core Laboratory of NHRI for histological examination.

### Statistical analysis

Data are presented as the mean ± SEM from at least three independent experiments. Statistical significance was determined by the unpaired Student's *t*-test. *P* < 0.05 was considered statistically significant. Graphs were prepared with GraphPad Prism (GraphPad Software v.8.0.1).

## Results

### Adjuvant activities of CpG-2722 and STING agonist combinations for intramuscularly and intranasally administrated SARS-CoV-2 RBD vaccine

CpG-2722 is a CpG-ODN with 19 nucleotides containing two GTCGTT hexamer CpG-motifs and four thymidine bases between these two motifs (Figure S1A) 33. The STING agonist 2'2'-cGAMP is a synthetic CDN containing two 2'-5'-phosphodiester bonds between the guanosine and adenosine, and 2'3'-cGAMP is a mammalian CDN containing 2'-5' and 3'-5' phosphodiester linkages between the guanosine and adenosine. 2'3'-cGAM(PS)2 is a bisphosphorothioated analog of 2'3'-cGAMP, and 2'3'-c-di-AM(PS)2 is a bisphosphorothioated analog of 2'3'-c-di-AMP (Figure S1B).

**Figure 1:**
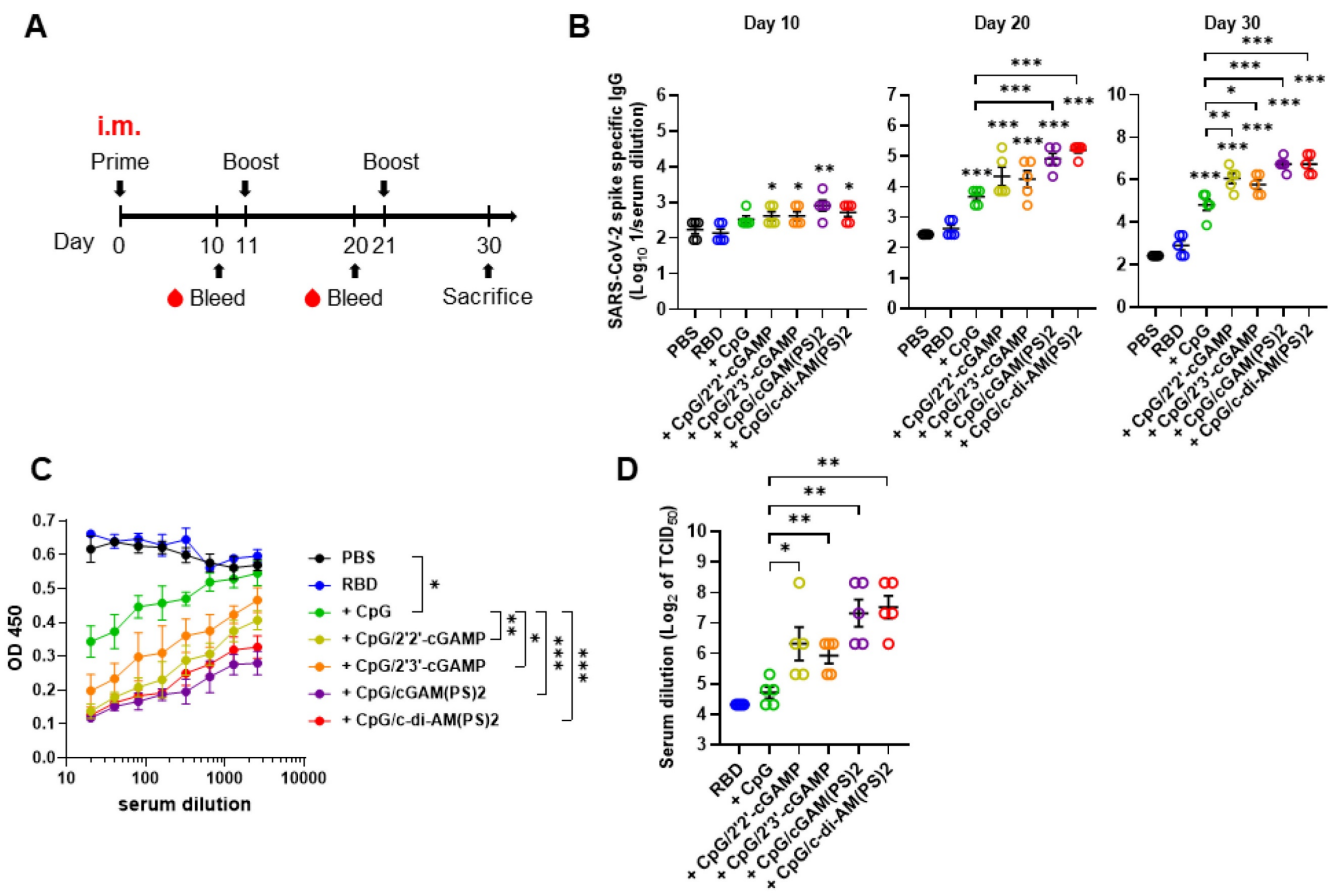
** RBD protein adjuvanted with combined CpG-2722/STING agonists induced a robust humoral response after intramuscular immunization. (A)** Schematic illustration of the experimental design. BALB/c mice were immunized intramuscularly on days 0, 11, and 21 with a vaccine formulated with 10 μg RBD protein, 10 μg CpG-2722, and 5 μg of different CDNs as indicated. **(B)** Serum samples were collected on days 10, 20, and 30, and the total amount of anti-S protein IgG was quantified with ELISA. Serum samples collected on day 30 were subjected to the hACE2-RBD competition assay** (C)** and the SARS-CoV-2 neutralization assay **(D)**. Data are the mean ± SEM (n = 5/group). *P < 0.05, **P < 0.01, and ***P < 0.001 compared with the group of mice treated with PBS vehicle control or between the indicated groups. CpG, cGAM(PS)2, and c-di-AM(PS)2 stand for CpG-2722, 2'3'-cGAM(PS)2 and 2'3'-c-di-AM(PS)2. + stands for plus RBD protein antigen.

To evaluate the adjuvant activities of combined CpG-2722 and CDNs, recombinant SARS-CoV-2 RBD protein was formulated as an antigen with/without CpG-2722 alone or in combination with different CDNs. BALB/c mice were intramuscularly immunized with these vaccines, and blood samples were collected in 10 days intervals to analyze antibody titers and anti-viral responses following the schedule in Figure 1A. As shown in Figure 1B, CpG-2722/CDN adjuvanted vaccines increased anti-S protein (hereafter anti-S) IgG titer on day 10 after the first inoculation. On day 20, after the 2^nd^ injection, CpG-2722 and CpG-2722/CDN combinations induced a significantly higher anti-S protein IgG titer than that induced by the vehicle control. Additionally, the CpG-2722/2'3'-cGAM(PS)2 and CpG-2722/2'3'-c-di-AM(PS)2 formulations enhanced anti-S IgG titer compared with CpG-2722 alone. These enhanced effects were also apparent for the CpG-2722/CDN formulations after the 3^rd^ vaccination (Figure 1B). Next, we analyzed the abilities of the serum samples from the immunized mice to block the interaction between RBD and ACE2 and neutralize the SARS-CoV-2 virus (hCoV-19/Taiwan/4/2020) infection in Vero E6 cells. Consistently, CpG-2722/CDN adjuvanted vaccines generated higher antibody titers for interfering with the RBD protein/ACE2 interaction and inhibition of viral infection than that generated by the vaccine adjuvanted with CpG-2722 alone (Figure 1C and D). The inhibition dilution of TCID_50_ (Median Tissue Culture Infectious Dose) for serum from the CpG-2722/2'2'-cGAMP, CpG-2722/2'3'-cGAMP, CpG-2722/2'3'-cGAM(PS)2, CpG-2722/2'3'-c-di-AM(PS)2 groups were 112, 64, 192, and 224, respectively (Figure 1D).

**Figure 2:**
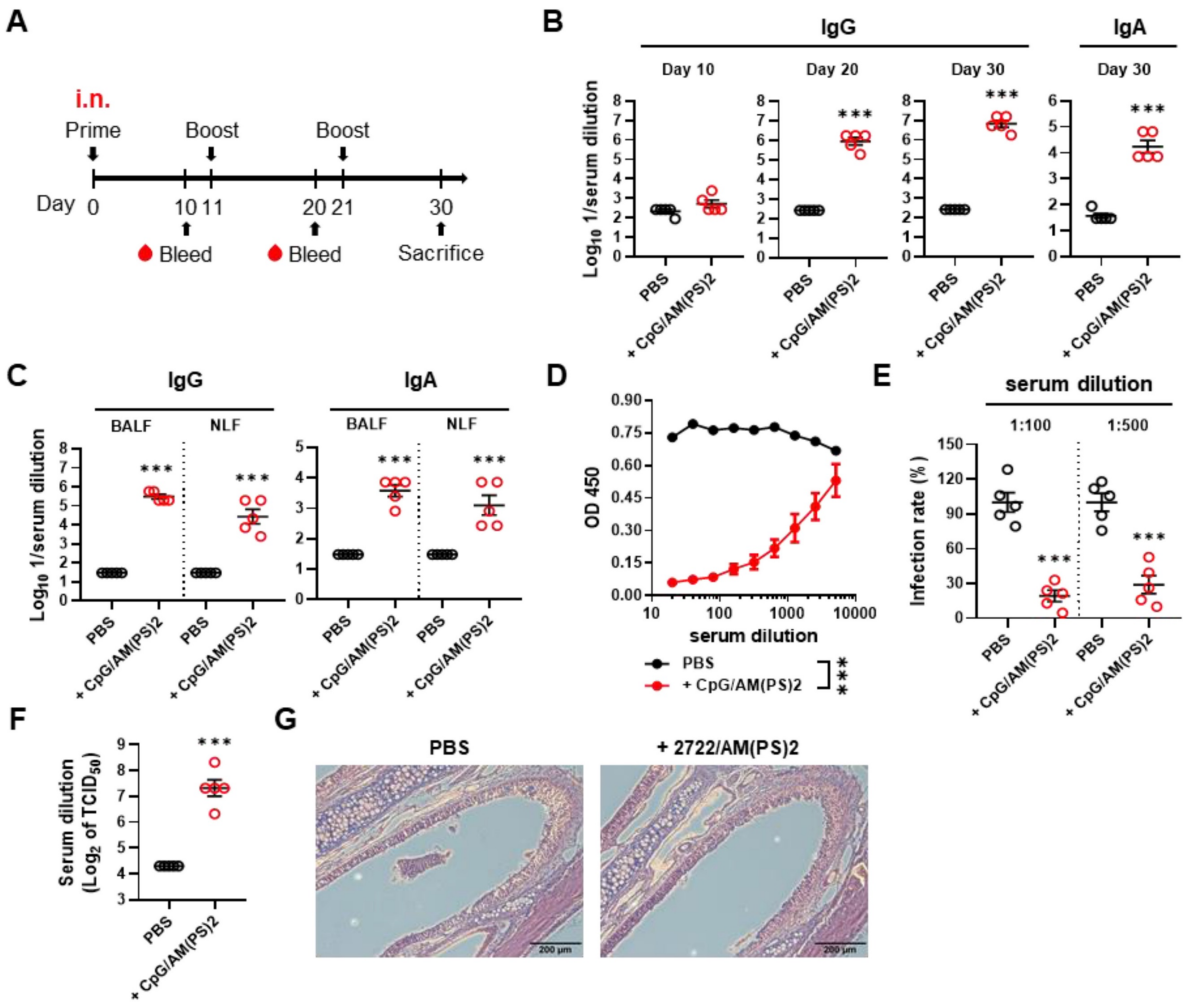
** RBD adjuvanted with CpG-2722/c-di-AM(PS)2 induced a robust humoral response via intranasal inoculation. (A)** Schematic illustration of the experimental design. BALB/c mice were immunized intranasally on days 0, 11, and 21 with a vaccine formulated with 10 μg of RBD protein, 10 μg CpG-2722, and 5 μg 2'3'c-di-AM(PS)2. Serum samples were collected on days 10, 20, and 30. The BALF, NLF, and nasal tissue of the vaccinated mice were collected 10 days after vaccination (day 30). The total amount of anti-S IgG and IgA from serum **(B)**, BALF, and NLF **(C)** were quantified with ELISA. Serum samples collected on day 30 were subjected to the hACE2-RBD competition assay **(D)**, a S protein-containing VSV pseudovirus neutralization assay **(E)**, and the SARS-CoV-2 neutralization assay **(F)**. **(G)** Representative H&E stained nasal tissues at 10 days post-challenge are shown. Data are the mean ± SEM (n = 5/group). ***P < 0.001 compared with mice treated with PBS vehicle control or between the different groups as indicated. CpG and AM(PS)2 stand for CpG-2722 and 2'3'-c-di-AM(PS)2. + stands for plus RBD protein antigen.

The adjuvant activity of the CpG-2722/2'3'-c-di-AM(PS)2 combination for intranasal immunization was investigated with a 10-days interval of nasal administration and blood sample collection schedule (Figure 2A). The CpG-2722 and 2'3'-c-di-AM(PS)2 combination robustly induced an antibody response to the SARS-CoV-2 S protein. Anti-S IgG was detected in the 10^6^-fold diluted serum collected after the second immunization. Furthermore, anti-S IgG and IgA were detected in the 10^7^ and 10^4^-fold diluted serum on day 30, respectively (Figure 2B). The levels of anti-S IgG and IgA in BALF and NLF after the third immunization were also investigated. Consistently, the CpG-2722/2'3'-c-di-AM(PS)2 adjuvanted vaccine generated considerable amounts of S protein-specific IgG and IgA in the bronchoalveolar and nasal cavities (Figure 2C). The collected sera from the immunized mice contained RBD neutralization antibodies to block the interaction between RBD and ACE2 (Figure 2D). The capability of these sera to interfere with the entrance of the S protein carrying vesicular stomatitis virus (S-VSV) pseudoviruses into hACE2-expressing BHK21 cells was investigated. The results revealed that 80.9% and 69.2% of the pseudovirus were inhibited by sera dilutions of 100 and 500, respectively (Figure 2E). Furthermore, in the SARS-CoV-2 neutralization assay, the sera showed an inhibition dilution of 176 for TCID_50_ (Figure 2F). Following immunizations, the mouse nasal cavities were histologically examined. No indication of pathological tissue damage or inflammation was found (Figure 2G). Taken together, these results demonstrate the potential of the CpG-2722/2'3'-c-di-AM(PS)2 combination as an adjuvant for muscle and nasal-delivered vaccines.

### Induction of germinal center B cell response by combinations of CpG-2722 and different STING agonists

**Figure 3:**
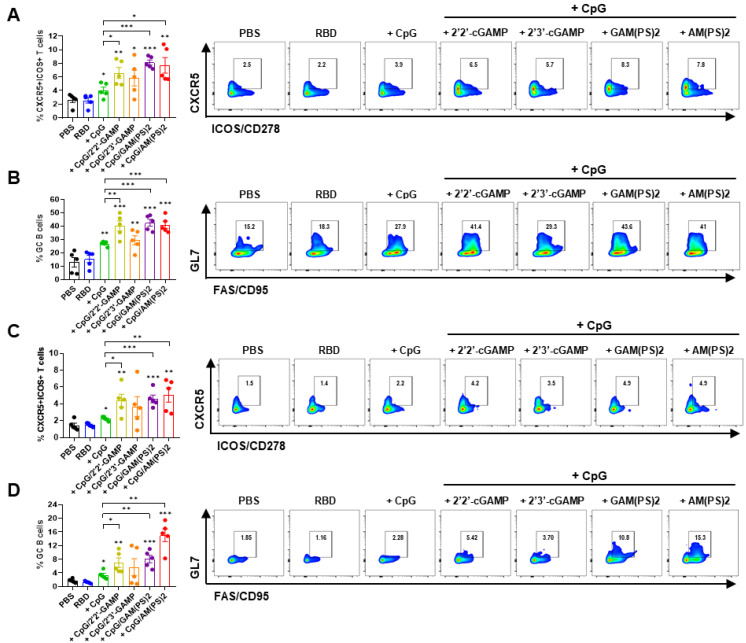
** A combination of CpG-2722 and STING ligands elicited germinal center (GC) T follicular helper (Tfh) cell and B cell responses.** In the experiment of Figure 1, the mice were euthanized 10 days after the final vaccination (day 30) for the collection of draining lymph nodes and spleens. The numbers of Tfh cells (CD4+Bcl-6+CXCR5+ICOS+) and GC B cells (B220+CO19+CD38-GL7+FAS+) from lymph nodes (A, B) and spleens (C, D) were measured by flow cytometry. Data are the mean ± SEM (n = 5/group). *P < 0.05, **P < 0.01, and ***P < 0.001 between the different groups as indicated. CpG, GAM(PS)2, and AM(PS)2 stand for CpG-2722, 2'3'-cGAM(PS)2 and 2'3'-c-di-AM(PS)2. + stands for plus RBD protein antigen.

Germinal center (GC) response is critical for the generation of mature plasma cells and memory B cells for long-lasting protective immunity. T follicular helper (Tfh) cells play a key role in regulating the GC response 35, 36. To assess the effects of CpG-2722/CDN combined adjuvants on the induction of a GC response, the vaccinated mice in the experiment for Figure 1 were euthanized after the final serum collection. Draining lymph node (dLN) cells and splenocytes were isolated for flow cytometry analysis of the CXCR5^+^/ICOS^+^ and CXCR5^+^/ PD-1^+^ Tfh cells and the GL7^+^/CD95^+^ GC B cells 37-39. The results showed that the CpG-2722/2'2'-cGAMP, CpG-2722/2'3'-cGAM(PS)2, and CpG-2722/2'3'-c-di-AM(PS)2 adjuvanted vaccines increased the Tfh and GC B cell counts in dLNs and spleens compared to the CpG-2722-adjuvanted vaccine (Figure 3 and Figure S2). Additionally, except for the CpG-2722/2'3'-cGAMP combination, the activities of these CpG-2722/CDN combinations on the induction of a GC B cell response were consistent with the humoral response shown in Figure 1.

### CpG-2722 and c-di-AM(PS)2 cooperatively boost immune responses to the SARS-CoV-2 RBD vaccine

**Figure 4:**
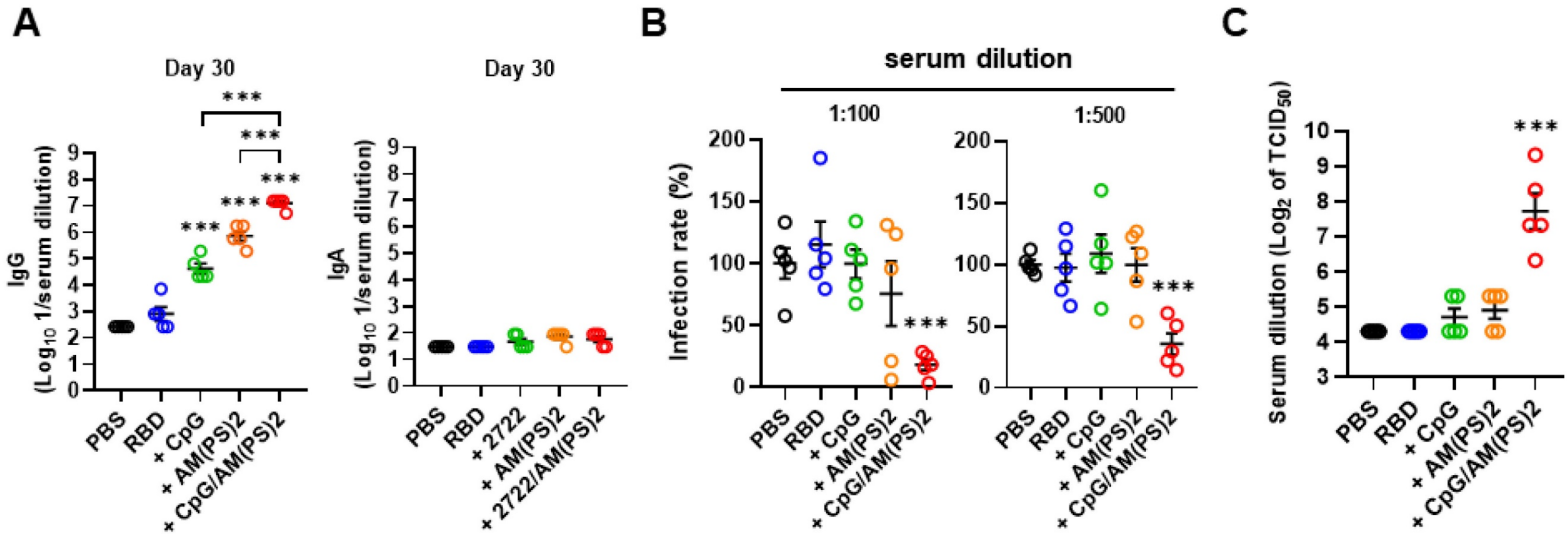
** Cooperative adjuvant effect of CpG-2722 and c-di-AM(PS)2 on inducing a humoral response to the RBD protein vaccine.** BALB/c mice were immunized intramuscularly on days 0, 11, and 21 with 10 μg of the RBD protein vaccine adjuvanted with 10 μg CpG-2722 and 5 μg c-di-AM(PS)2 alone or in combination. Serum samples were collected on days 30. **(A)** The total amount of anti-S protein IgG (left panel) and IgA (right panel) were quantified with ELISA. **(B)** These samples were subjected to the S-containing VSV pseudovirus neutralization assay, and **(C)** the SARS-CoV-2 neutralization assay. Data are the mean ± SEM (n = 5/group). *P < 0.05, **P < 0.01, and ***P < 0.001 compared with mice treated with PBS vehicle control or between the different groups as indicated. CpG and AM(PS)2 stand for CpG-2722 and 2'3'-c-di-AM(PS)2. + stands for plus RBD protein antigen.

2'3'-c-di-AM(PS)2 (hereafter c-di-AM(PS)2) was used to study the molecular basis for the adjuvant activity of the CpG-2722 and STING agonist combination. Whether the increased adjuvant effect of CpG-2722/c-di-AM(PS)2 came from a cooperative effect of these two agonists was first investigated. SARS-CoV-2 RBD protein was formulated with or without CpG-2722 and c-di-AM(PS)2 alone or in combination. Mice were vaccinated with a 10-day interval intramuscular immunization and blood collection schedule, as shown in Figure 1A. On day 10 after the first inoculation, anti-S IgG was detected in the group that received the CpG-2722/c-di-AM(PS)2 adjuvanted vaccine, but not in the groups that received the CpG-2722 or c-di-AM(PS)2 alone adjuvanted vaccine (Figure S3A). On day 20, after the 2^nd^ injection, the vaccine adjuvant activities of CpG-2722 and c-di-AM(PS)2 became apparent, and the CpG-2722/c-di-AM(PS)2 combination induced a significantly higher anti-S protein IgG titer than that induced by the CpG-2722 or c-di-AM(PS)2 alone (Figure S3B). On day 30, after completing the entire immunization process, the immune sera from different groups were examined for the levels of anti-S IgG and anti-S IgA. The vaccine adjuvanted with the CpG-2722/c-di-AM(PS)2 combination continued to induce a higher anti-S IgG titer than that induced by the CpG-2722 or the c-di-AM(PS)2 adjuvanted vaccines. In contrast, the vaccines adjuvanted with CpG-2722 and c-di-AM(PS)2 alone or in combination did not elicit the production of serum anti-S IgA (Figure 4A). This was different from that seen in Figure 2B for the induction of anti-S IgA by the intranasally administrated vaccine. These sera from different adjuvanted vaccine groups contained neutralization antibodies that blocked the binding of RBD protein to hACE2. Moreover, sera from the CpG-2722/c-di-AM(PS)2 adjuvanted group had a stronger blocking activity than that from the CpG-2722 or the c-di-AM(PS)2 alone adjuvanted groups (Figure S3C). The capability of these sera to interfere with the entrance of S-VSV pseudoviruses into hACE2-expressing BHK21 cells and to neutralize infection with SARS-CoV-2 viruses into Vero cells were investigated. The results revealed 81.9% and 71.8% blocking of the pseudovirus infection by the sera from the CpG-2722/c-di-AM(PS)2 adjuvanted vaccine group at 100 and 500-fold dilution, respectively (Figure 4B). Additionally, in the SARS-CoV-2 neutralization assay, the sera showed an inhibition dilution of 208 for TCID_50_ (Figure 4C). Furthermore, in both studies, sera from the CpG-2722/c-di-AM(PS)2 adjuvanted vaccine group showed stronger neutralization activity compared to that of the CpG-2722 or the c-di-AM(PS)2 adjuvanted vaccine groups (Figure 4B and C). These results suggest a cooperative adjuvant effect between the TLR9 and the STING agonists on boosting the humoral immune response to the vaccine.

### Antigen-dependent T helper responses induced by vaccines adjuvanted with CpG-2722 and c-di-AM(PS)2 alone and in combination

**Figure 5:**
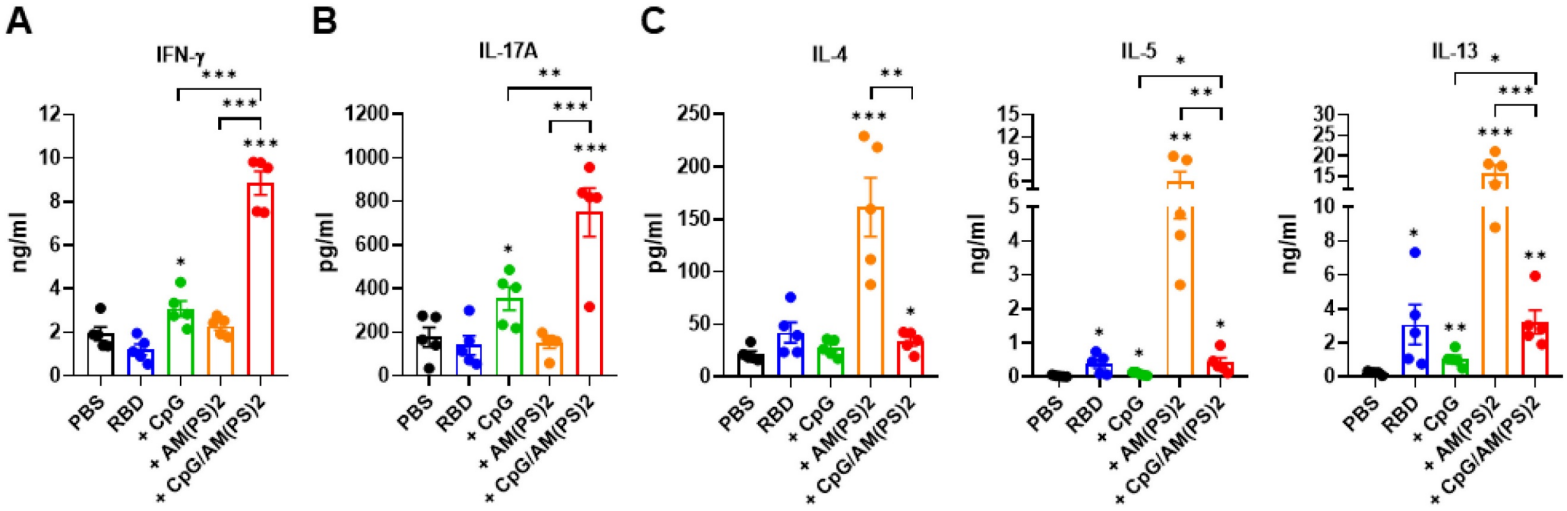
** Antigen-dependent T helper responses in mice immunized with RBD protein vaccines adjuvanted with CpG-2722 and c-di-AM(PS)2 alone or in combination.** In the experiments of Figure 4, mice were euthanized 10 days after the final immunization. **(A-C)** Splenocytes were prepared and stimulated with RBD for 96 h. The concentrations of signature cytokines for Th1 (IFN-γ) **(A)**, Th17 (IL-17A) **(B)**, and Th2 (IL-4, IL-5, IL-13) **(C)** in the culture supernatants were measured by ELISA. Data are the mean ± SEM (n = 5/group). *P < 0.05, **P < 0.01, and ***P < 0.001 compared with mice treated with PBS vehicle control or between the different groups as indicated. CpG and AM(PS)2 stand for CpG-2722 and 2'3'-c-di-AM(PS)2. + stands for plus RBD protein antigen.

At the endpoint of the experiment in Figure 4, splenocytes from a different group of mice were collected to investigate antigen-dependent Th responses. These cells were stimulated with the RBD protein antigen and the production of signature cytokines for different Th responses were measured with ELISA. These analyzed cytokines were: IFN-γ for Th1, IL-17A for Th17, and IL-4, IL-5, and IL-13 for Th2 response 40, 41. The results showed that CpG-2722 adjuvanted vaccine preferentially induced antigen-dependent Th1 and Th17 responses in the immunized mice, whereas the c-di-AM(PS)2 adjuvanted vaccine induced an antigen-dependent Th2 response. Furthermore, compared to that induced by the CpG-2722 and c-di-AM(PS)2 adjuvanted vaccines, combining CpG-2722 and c-di-AM(PS)2 in a vaccine reshaped the Th response profile in which the Th1 and Th17 responses were synergistically enhanced and the Th2 response was suppressed but compared to the vehicle control, remained significantly activated (Figure 5).

### Activation of dendritic cells by CpG-2722, c-di-AM(PS)2 alone and their combination

**Figure 6:**
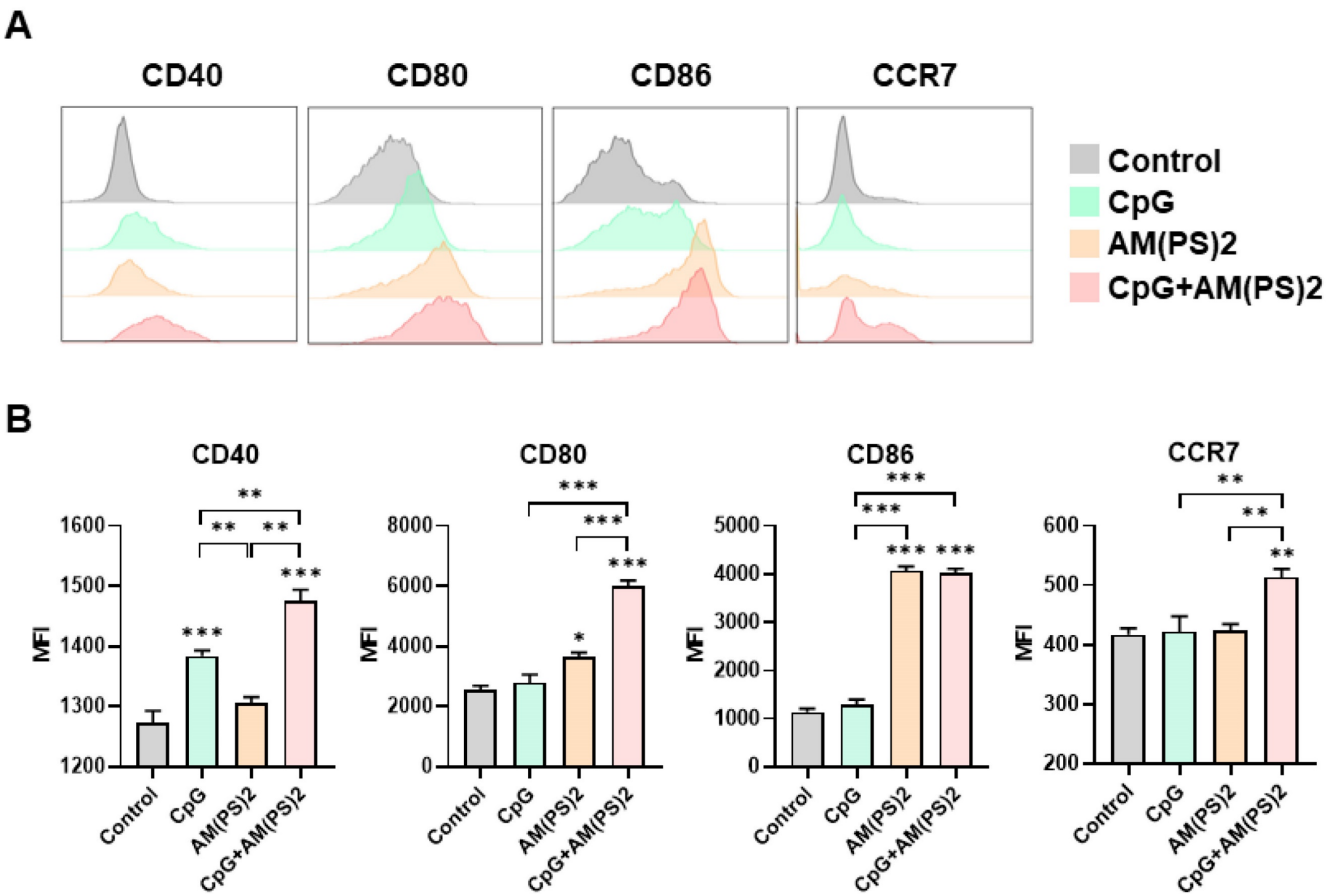
** CpG-2722 and c-di-AM(PS)2 cooperatively promoted the activation of BMDCs.** Mouse BMDCs were stimulated with CpG-2722 (5 μg/ml), c-di-AM(PS)2 (1 μg/ml), or a combination of both. After 24 h, cells were collected and analyzed the surface expression of CD11c, CD40, CD80, CD86, and CCR7 by flow cytometry. **(A)** Representative histograms were shown. **(B)** The MFI of CD40+, CD80+, CD86+, and CCR7+ cells in a population of CD11c+ cells were quantified. Data are the mean ± SEM (n = 4). *P < 0.05, **P < 0.01, and ***P < 0.001 compared with control cells or between the different groups as indicated. CpG and AM(PS)2 stand for CpG-2722 and 2'3'-c-di-AM(PS)2.

**Figure 7:**
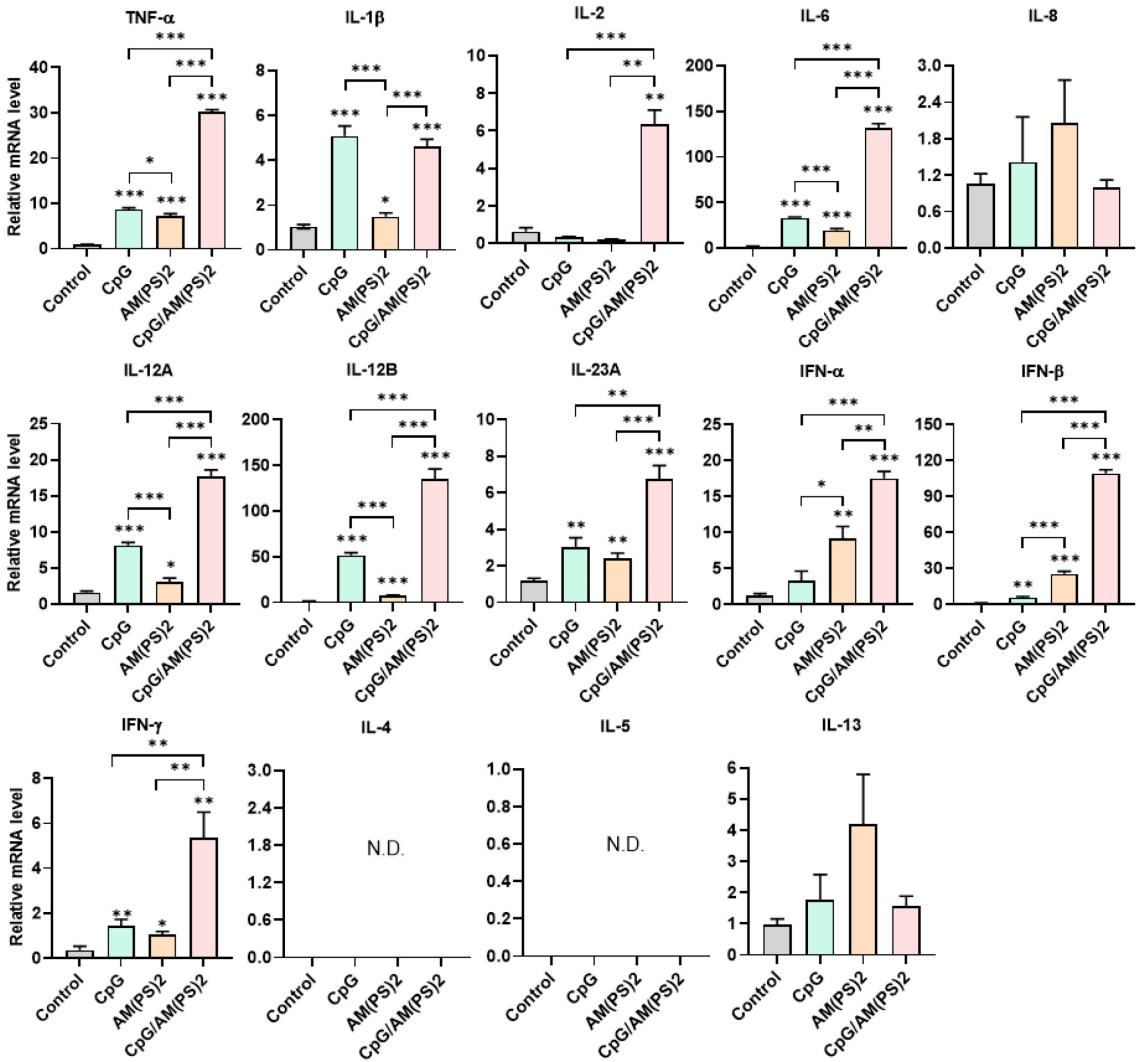
** Effect of CpG-2722, c-di-AM(PS)2, and their combination on cytokine expression in mouse BMDCs.** Mouse BMDCs were cultured with CpG-2722 (5 μg/ml), c-di-AM(PS)2 (1 μg/ml), or a combination of both. After 4 h, the total RNA was isolated. The expression of different cytokines in the cells was determined by RT-qPCR and normalized to β-actin levels. Data are presented as fold induction compared with the mRNA level in PBS vehicle control treated cells. Data are the mean ± SEM (n = 4). *P < 0.05, **P < 0.01, and ***P < 0.001 compared with control cells or between the different groups as indicated. CpG and AM(PS)2 stand for CpG-2722 and 2'3'-c-di-AM(PS)2.

Dendritic cells (DCs) are professional antigen-presenting cells and their activation for antigen presentation and cytokine production is critical for effective vaccination and adjuvant-activated antigen-dependent T cell responses 42, 43. Therefore, we investigated the capability of CpG-2722, c-di-AM(PS)2, and their combination to induce the expression of cell surface molecules and cytokines that serve various functions in the activation and maturation of DCs. The expressions of cell surface molecules on bone marrow-derived dendritic cells (BMDCs) were analyzed by flow cytometry following different treatments. CpG-2722 alone induced expression of CD40, c-di-AM(PS)2 alone induced the expression of CD80 and CD86, while none of these two agonists activated expression of the CCR7. The combination of CpG-2722 and c-di-AM(PS)2 displayed a cooperative effect in increasing the expression of CD40, CD80, and CCR7, but the c-di-AM(PS)2 induced CD86 expression was not further increased when combined with CpG-2722 (Figure 6). CD40, CD80, and CD86 are markers for DC activation and maturation. They are co-stimulatory molecules that promote T cell activation. The CCR7 is a chemokine receptor that plays a critical role in DC migration to lymphoid organs to activate T cells 44, 45. The activities of CpG-2722, c-di-AM(PS)2, and their combination in inducing cytokine expression in BMDCs were investigated by RT-qPCR. Both CpG-2722 and c-di-AM(PS)2 alone induced the expressions of TNF-α, IL-1β, IL-6, IL-12A, IL-12B, IL-23A, IFN-β, and IFN-γ genes. c-di-AM(PS)2 alone induced the expression of IFN-α. Except for IL-1β, which was strongly induced by the CpG-2722, the combination of CpG-2722 and c-di-AM(PS)2 showed a cooperative effect on inducing the expression of all other cytokines. Notably, the CpG-2722/c-di-AM(PS)2 combination induced the expression of IL-2, which was not induced by either of these two agonists alone (Figure 7). IL-2 production in DCs has been shown to play an important role in DCs-derived T cell activation 42, 43.

### Antigen-independent cytokine-inducing profiles of CpG-2722 and c-di-AM(PS)2 alone and in combination in different cell types

**Figure 8:**
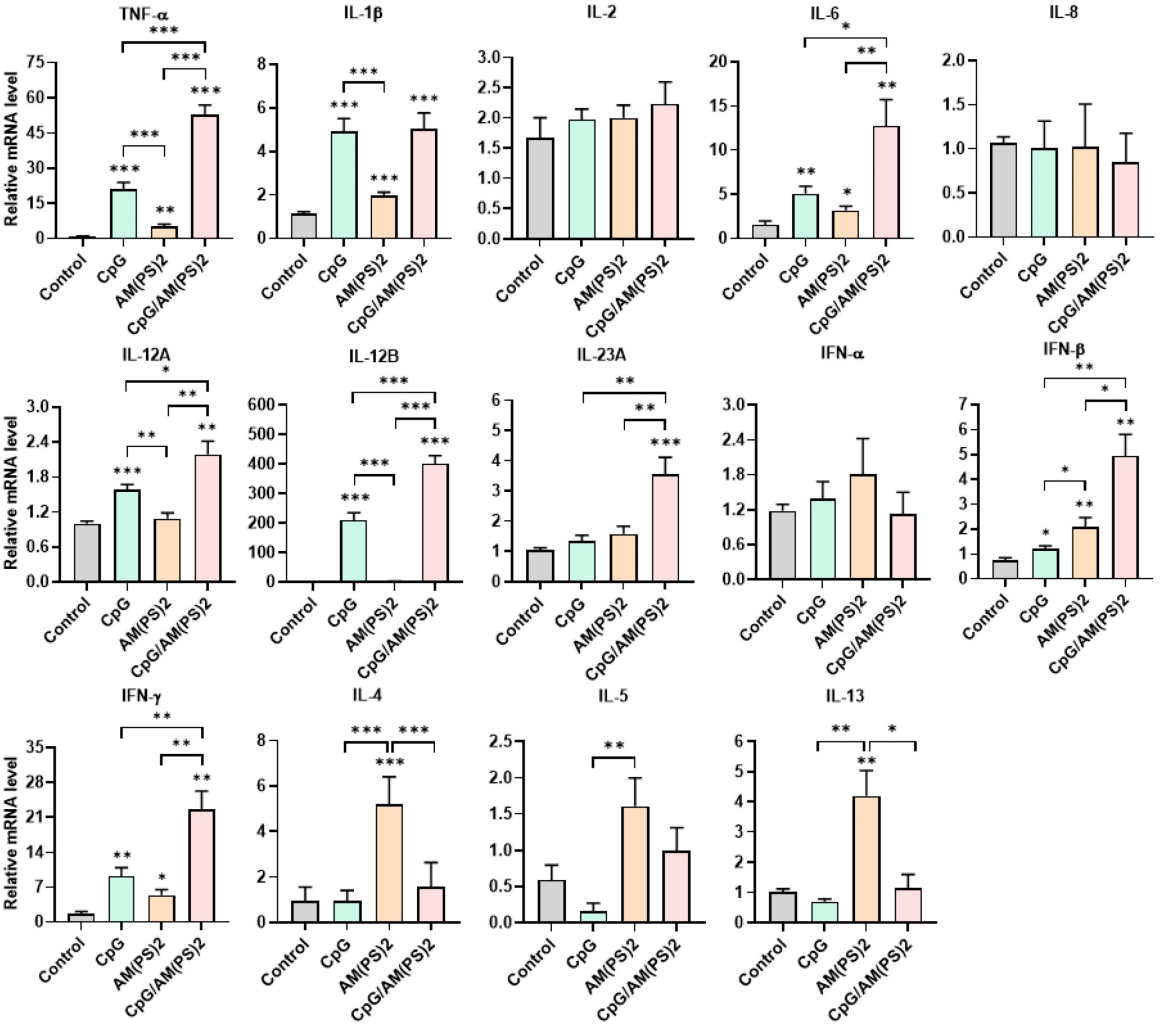
** Effect of CpG-2722, c-di-AM(PS)2, and their combination on cytokine expression in mouse splenocytes.** Mouse splenocytes were cultured with CpG-2722 (5 μg/ml), c-di-AM(PS)2 (1 μg/ml), or a combination of both. After 4 h, the total RNA was isolated. The expression of different cytokines in the cells was determined by RT-qPCR and normalized to β-actin levels. Data are presented as fold induction compared with the mRNA level in PBS vehicle control treated cells. Data are the mean ± SEM (n = 3). *P < 0.05, **P < 0.01, and ***P < 0.001 compared with control cells or between the different groups as indicated. CpG and AM(PS)2 stand for CpG-2722 and 2'3'-c-di-AM(PS)2.

**Figure 9:**
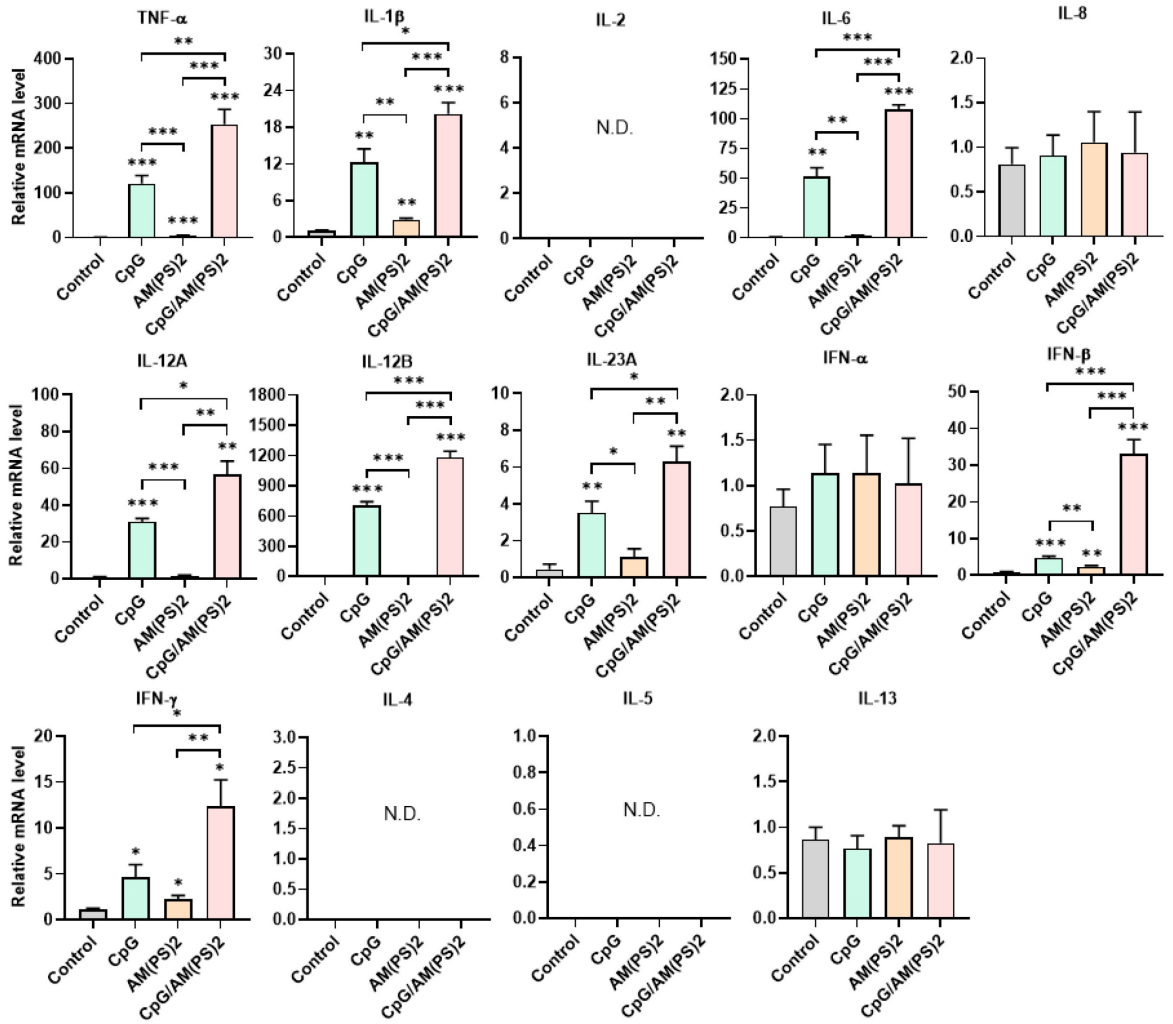
** Effect of CpG-2722, c-di-AM(PS)2, and their combination on cytokine expression in mouse PBMCs.** Mouse PBMCs were cultured with CpG-2722 (5 μg/ml), c-di-AM(PS)2 (1 μg/ml), or a combination of both. After 4 h, the total RNA was isolated. The expression of different cytokines in the cells was determined by RT-qPCR and normalized to β-actin levels. Data are presented as fold induction compared with the mRNA level in PBS vehicle control treated cells. Data are the mean ± SEM (n = 3). *P < 0.05, **P < 0.01, and ***P < 0.001 compared with control cells or between the different groups as indicated. CpG and AM(PS)2 stand for CpG-2722 and 2'3'-c-di-AM(PS)2.

**Figure 10:**
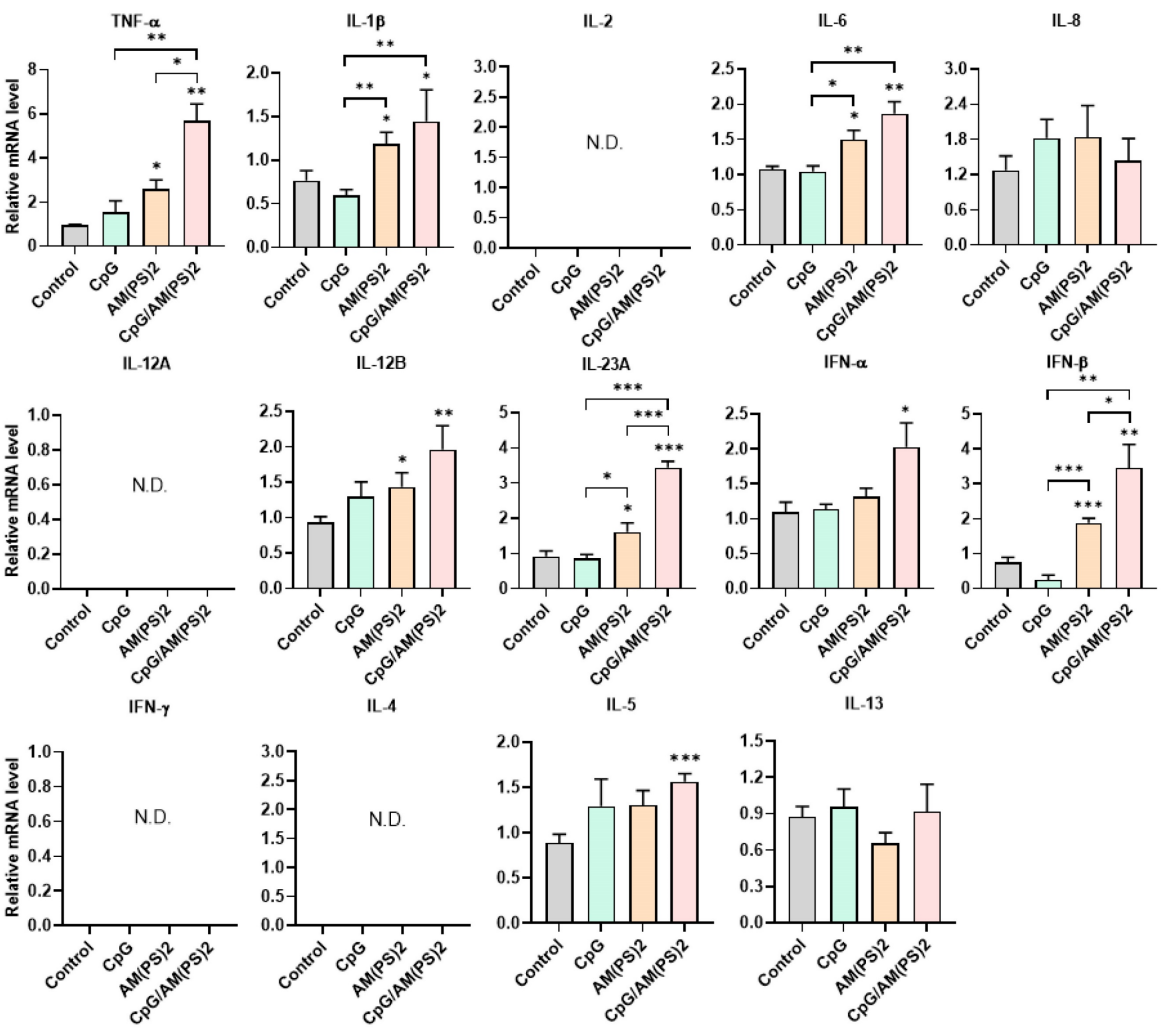
** Effect of CpG-2722 and c-di-AM(PS)2 on cytokine production in MEFs.** MEFs were cultured with CpG-2722 (5 μg/ml), c-di-AM(PS)2 (1 μg/ml), or a combination of both. After 4 h, the total RNA was isolated. The expression of different cytokines in the cells was determined by RT-qPCR and normalized to β-actin levels. Data are presented as fold induction compared with the mRNA level in PBS vehicle control treated cells. Data are the mean ± SEM (n = 3). *P < 0.05, **P < 0.01, and ***P < 0.001 compared with control cells or between the different groups as indicated. CpG and AM(PS)2 stand for CpG-2722 and 2'3'-c-di-AM(PS)2.

To further gain insight into the mechanism by which antigen-dependent Th responses are shaped by different adjuvants in immunized mice, the activities of CpG-2722, c-di-AM(PS)2 alone and in combination to induce the expression of cytokines in different cell populations were investigated. In splenocytes, CpG-2722 was more potent than c-di-AM(PS)2 in inducing the expression of TNF-α, IL-1β, IL-6, IL-12 A, IL-12B, and IFN-γ. Among these cytokines, IL-12A and IL-12B were not induced by c-di-AM(PS)2 alone. In contrast, c-di-AM(PS)2 induced the expression of IFN-β, IL-4, IL-5, and IL-13 more potently. Furthermore, the combination of CpG-2722 and c-di-AM(PS)2 had a cooperative effect on inducing the expression of TNF-α, IL-6, IL-12A, IL-12B, IL-23A, IFN-β, and IFN-γ, but the expression of IL-4, IL-5, and IL-13 were suppressed with this combination (Figure 8). Like in splenocytes, the mRNA expression of TNF-α, IL-1β, IL-6, IL-12A, IL-12B, IL-23A, IFN-β as well as IFN-γ in PBMCs showed a higher induction by CpG-2722 treatment alone than that induced by the c-di-AM(PS)2 treatment alone. The combination of these agonists had a cooperative effect on inducing the expression of these cytokines. However, in PBMCs, the expression levels of IL-2, IL-4, and IL-5 were too low to be detected, and the expression of IL-13 was not increased by any stimulation (Figure 9). In MEFs, cytokine expression activation by CpG-2722 was not observed. In contrast, c-di-AM(PS)2 or the CpG-2722/c-di-AM(PS)2 combination induced the gene expression of TNF-α, IL-1β, IL-6, IL-12B, IL-23A, and IFN-β. Among these genes, TNF-α, IL-23A, and IFN-β expression was further enhanced with CpG-2722/c-di-AM(PS)2 combinational stimulation compared with c-di-AM(PS)2 alone (Figure 10).

**Figure 11:**
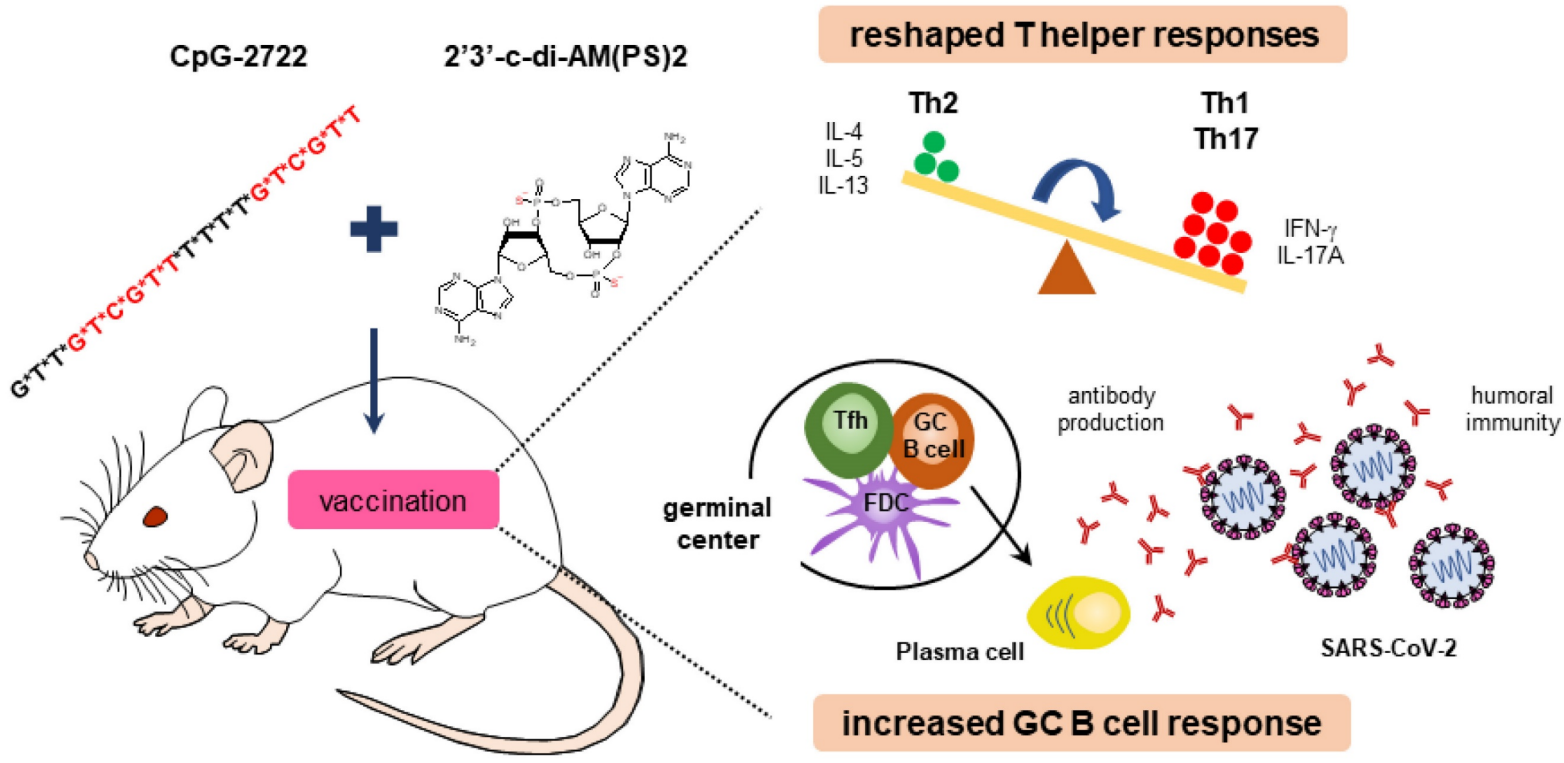
** Cooperative adjuvant activity of TLR9 agonist and STING agonist combination.** CpG-2722 a TLR9 agonist and c-di-AM(PS)2 a STING agonist cooperatively boost the immune response to SARS-CoV-2 RBD vaccine through an increased germinal center B cell response and reshaped T helper responses.

These results obtained from BMDCs, splenocytes, PBMCs, and MEFs show that CpG-2722 and c-di-AM(PS)2 have different targeting cell populations and thus have different cytokine-inducing profiles in different cell populations. TNF-α and IFN-γ induced by CpG-2722 are required for Th1 cell differentiation and the activation of IL-1, IL-6, and IL-23 are essential for Th17 cell development. In contrast, c-di-AM(PS)2 preferentially induces IL-4, which plays a key role in the activation of Th2 cell differentiation 39, 40. These antigen-independent responses shaped the antigen-dependent responses induced by the vaccines adjuvanted with CpG-2722 and c-di-AM(PS)2 alone or in combination. Overall, the increased targeting cell populations, enhanced germinal center B cell response, and the reshaped T helper responses are the molecular bases for the cooperative adjuvant effect of the combination of TLR9 and STING agonists (Figure 11).

## Discussion

Vaccines are a powerful tool for preventing epidemic diseases by controlling the spread of microbial infections. Efficient vaccines typically rely on an effective adjuvant to boost immune system responses. Since the discovery of aluminum salts as a vaccine adjuvant in the 1920s, progress in the development of adjuvants has been relatively slow. Although research has increased in the last decade, fewer than fifteen different adjuvants have been used in vaccines for humans 13, 46, 47. Therefore, there is a need to continuously explore adjuvant candidates for the development of efficacious vaccines. In this study, we used the SARS-CoV-2 RBD protein as an antigen to investigate the activity and the molecular basis for the function of an adjuvant composed of TLR9 and STING agonists.

TLR9 has a narrow cell expression profile. It is mainly expressed in dendritic cells and B cells and to some extent in monocytes/macrophages, neutrophils, and T cells 48, 49. Synthetic TLR9 agonists, CpG-ODNs have been investigated as vaccine adjuvants for a long time. CpG-1018 has been formulated as an adjuvant in Heplisav-B, which is a hepatitis B vaccine approved by the FDA in 2017. Additionally, MVC-COV1901 and Corbevax are formulated with CpG-1018, and Advax-CpG55.2 is formulated with CpG-55.2. These vaccines are EUA for SARS-CoV-2 in at least one country 24-27. Other than these, different CpG-ODNs have been or are being investigated as vaccine adjuvants for various infectious diseases 13, 18. In this study, we showed that a CpG-2722-formulated vaccine promoted an increase in Tfh and GC B cell count. This GC B cell response is required for the development of B cells into antibody-secreting plasma cells or memory B cells, which are long-lived B cell populations and are essential for a high-quality protective vaccine effect 35, 36, 50, 51. Additionally, the CpG-2722-formulated vaccine raised antigen-dependent Th1 and Th17 responses in immunized mice. CpG-2722 also induced the expression of various cytokines in different cell types. Of these cytokines, TNF-α and IFN-γ promote Th1 cell differentiation, and IL-1, IL-6, and IL-23 promote Th17 cell development 40, 41. These explain the antigen-dependent Th1 and Th17 responses elicited by the adjuvant effect of CpG-2722. CpG-ODNs usually contain species-specific activity, which is determined by the nucleotide context of its CpG-hexamer motifs. CpG-ODN with a GTCGTT-hexamer motif is a more potent activator in human cells than in murine cells. In contrast, CpG-ODN with a GACGTT-hexamer motif is preferred for the activation of murine cells. The CpG-2722 used in this study contains 19 nucleotide bases and two GTCGTT-hexamer motifs; nevertheless, it has been shown to have immunostimulatory activities in different species 33, 52, 53. Thus, the function of CpG-2722 observed in this study is likely to be replicated in humans.

Compared to TLR9, STING has an overlapped but distinct cell distribution pattern. It is expressed in immune cells including dendritic cells, macrophages, natural killer cells, and T cells, as well as in non-immune cells such as endothelial cells, epithelial cells, and fibroblasts 54, 55. In line with this, the immune stimulatory activity of c-di-AM(PS)2 but not CpG-2722 was seen in MEFs. STING agonists including various CDNs are being intensively investigated for application as adjuvants for various vaccines. Nevertheless, most of these studies are in the preclinical stages. STING agonist has not been used in licensed vaccines 20, 31, 32. The effects of different CDNs on inducing Th responses have not yet been consistent in past studies. c-di-GMP induced a Th1 response, including strong induction of IFN-γ, TNF-α, IL-12, and IP-10 in non-human primate PBMCs. When formulated as an adjuvant, it induced more predominant GC formation and a humoral immune response in immunized mice than that in mice immunized with LPS or CpG-ODN-adjuvanted vaccines 56. In mice, intranasal immunization with chitosan and c-di-GMP together adjuvanted influenza H5N1 vaccine, c-di-GMP induced antigen-specific CD4^+^ T cells producing Th1 cytokines 57. Similarly, immunization of mice with c-di-AMP-adjuvanted ovalbumin induced a strong Th1 response and antigen-dependent immune responses compared to mice immunized with poly(I:C)/CpG-ODN-adjuvanted ovalbumin 58. In contrast, in mice with β-galactosidase as a model antigen, c-di-GMP and c-di-AMP were reported to induce Th1/Th2 and Th1/Th2/Th17 balanced immune responses, respectively 59, 60. Additionally, c-di-IMP induced a mixed Th1/Th2/Th17 response, and cGAMP induced a balanced Th1/Th2 response in mice when used as a mucosal adjuvant 61, 62. In this study, we showed that the c-di-AM(PS)2 adjuvanted SARS-CoV-2 RBD protein vaccine induced an antigen-dependent Th2 response in immunized mice. c-di-AM(PS)2 induced expression of IL-4 in splenocytes. IL-4 is a key cytokine in driving Th2 cell differentiation. This explains the observed Th2 response in immunized mice. The induction of an IL-4-mediated Th2 immune response by a STING agonist has not yet been reported. Nevertheless, a previous study ever showed suppression of T-bet and the induction of GATA3 by the cGAS-STING pathway 63. GATA3 is a master transcription factor for Th2 differentiation and plays a role in the regulation of IL-4 expression 64, 65. Conversely, IL-4 regulates the expression of GATA3 66. These are likely to form a positive loop for the regulation of IL-4 production by the cGAS-STING pathway.

Some STING and TLR agonist combinations were investigated for cancer immunotherapy. Systemic delivery of c-di-GMP and monophosphoryl lipid A (a TLR4 agonist)-encapsulated nanoparticles generated a significant antitumor effect in a breast cancer animal model 67. Compared to single treatments, the combination of CpG-ODN and cGAMP together significantly inhibited tumor growth in mouse models of melanoma and lymphoma 68. The formulation of cGAMP and CpG-ODN together with a mutated form of the HPV16 E7 protein as a tumor vaccine led to the suppression of tumor growth in a mouse model 69. A combination of c-di-AMP and CpG-ODN was shown to generate cooperative antitumor immunity and a tumor-suppressing effect in a pancreatic cancer mouse model 70. The adjuvant function of the TLR9 and STING agonist combination for vaccines is little studied. In this study, we found that CpG-2722 and c-di-AM(PS)2 together enhanced the CpG-2722-promoted GC response and antigen-dependent Th1 and Th17 responses. Furthermore, this combination suppressed the c-di-AM(PS)2-induced antigen-dependent Th2 response. This resulted in a reshaped Th response profile characterized by high Th1 and Th17, but low Th2, responses. The CpG-2722 and c-di-AM(PS)2 combination cooperatively activated surface expression of CD40, CD80 and CCR7 on DCs, which are key molecules for dendritic cell mediated T cell activation. The generated antigen-dependent Th responses were shaped by the cytokines induced by the CpG-2722 and c-di-AM(PS)2 alone and in combination in an antigen-independent manner. As seen in the splenocytes, BMDCs, PBMCs, or MEFs, both CpG-2722 and c-di-AM(PS)2 are able to induce the expression of TNF-α, IFN-γ , IL-6, and IL-23 and induction of these cytokines are cooperatively enhanced by combination of these two agonists. These cytokines are required for Th1 and Th17 cell differentiations, thus could explain the enhanced Th1 and Th17 induced by the combination. IFN-γ produced in the Th1 response has been shown to diminish the IL-4 production required for a Th2 response 71, 72. CpG-2722 potently induced the expression of IFN-γ. Thus, it is reasonable to observe a suppressive effect of CpG-2722 on the c-di-AM(PS)2-induced Th2 response.

Th1 and Th17 immune responses are usually generated in response to viral infections and TLR stimulation. IFN-γ, the signature cytokine of Th1, can promote a humoral response to viral infection by controlling the immunoglobulin isotypes produced by B cells. Th17 cells can also function as B cell helpers to induce B cell proliferation and antibody production 40, 41, 73, 74. Although Th2 cytokines including IL-4, IL-5, and IL-13 also play roles in the development of the humoral immune response by activating B cell proliferation and antibody production, most of the licensed aluminum-adjuvanted vaccines elicit a Th2 response, nevertheless, the adjuvant effect is usually weak, and repeated immunizations are often required 75, 76. Recently, rational design by mixing different immune stimuli to generate an effective adjuvant usually emphasizes a mixed Th1/Th2/Th17 response. However, how the magnitude of each Th response is adjusted to reach an optimized response profile remains unclear 77, 78. In this study, we showed that a Th1 high/Th17 high/Th2 low profile generated by the combination of CpG-2722 and c-di-AM(PS)2 has a superior adjuvant effect than the Th1/Th17 effect generated by CpG-2722 alone and the Th2 effect generated by c-di-AM(PS)2 alone. Whether such a profile of mixed Th responses is a preferred profile for a combined adjuvant remains for further study.

This study not only demonstrated the cooperative adjuvant activity of the combination of CpG-2722 and c-di-AM(PS)2 in an intramuscular vaccine but also exhibited a superior adjuvant effect of this combination in an intranasal SARS-CoV-2 RBD vaccine. Nasal immunization, in addition to inducing systemic immunity, generally elicits strong mucosal immunity, including the production of antigen-specific IgA, which is favorable for combating respiratory infections 79, 80. Accordingly, the CpG-2722 and c-di-AM(PS)2 combination adjuvant induced a significant amount of antigen-specific IgA and IgG when administered nasally, while only IgG was induced by the intramuscularly administered vaccine. Nasal vaccines have several advantages, such as being less invasive and more easily accepted by most people, especially children, who constitute a significant population for vaccination. However, the development of nasal vaccines lags behind compared to injected vaccines. Currently, only one nasal vaccine, FluMist, has been approved by the US FDA for influenza. The major challenges in developing nasal vaccines are antigenicity and safety issues. Approaches to overcome these obstacles include the development of safe and effective adjuvants 79, 80. In this regard, it would be of interest to investigate further the potential of developing TLR9 and STING agonist combinations to be used as adjuvants for nasal vaccines. In summary, this study showed that a combination of TLR9 and STING agonists as adjuvants, effectively increased the immune response to intramuscularly and intranasally administrated SARS-CoV-2 RBD protein vaccine compared to using either of these two agonists alone. The underlying mechanism involves broader targeting of cell populations by the combined adjuvant, an increased GC B cell response, and reshaped Th responses due to the combination.

## Supplementary Material

Supplementary figures and table.
