# Supplementary material for: TLR9 and STING agonists cooperatively boost the immune response to SARS-CoV-2 RBD vaccine through an increased germinal center B cell response and reshaped T helper responses
Source: Int J Biol Sci. 2023 May 29;19(9):2897–913. doi: 10.7150/ijbs.81210 (PMC10266083; doi:10.7150/ijbs.81210)
Supplement: Supplementary file 1 — Supplementary figures and table. [file ijbsv19p2897s1.pdf]

Table S1. List of qPCR primer sequences used in this study.

Gene	Forward	Reverse
IL-1β	AGAGCTTCAGGCAGGCAGTA	AGGTGCTCATGTCCTCATCC
IL-6	CCGGAGAGGAGACTTCACAG	TTTCCACGATTTCCCAGAGA
IL-8	CATTTGGGAGACCTGAGAACA	TGGAGTCCCGTAGAAAATTCC
IL-12A	ACGGCCAGAGAAAAACTGAA	CTACCAAGGCACAGGGTCAT
IL-12B	CACGCCTGAAGAAGATGACA	AGTCCCTTTGGTCCAGTGTG
IL-23A	CATGCTAGCCTGGAACGCACAT	ACTGGCTGTTGTCCTTGAGTCC
TNF-α	ACGGCATGGATCTCAAAGAC	GTGGGTGAGGAGCACGTAG
IFN-α	ATCCAGAAGGCTCAAGCCATCC	GGAGGGTTGTATTCCAAGCAGC
IFN-β	GCCTTTGCCATCCAAGAGATGC	ACACTGTCTGCTGGTGGAGTTC
IFN-γ	CAGCAACAGCAAGGCGAAAAAGG	TTTCCGCTTCCTGAGGCTGGAT
IL-2	GCGGCATGTTCTGGATTTGACTC	CCACCACAGTTGCTGACTCATC
IL-4	ATCATCGGCATTTTGAACGAGGTC	ACCTTGGAAGCCCTACAGACGA
IL-5	GATGAGGCTTCCTGTCCCTACT	TGACAGGTTTTGGAATAGCATTTCC
IL-13	AACGGCAGCATGGTATGGAGTG	TGGGTCCTGTAGATGGCATTGC
β-Actin	CATTGCTGACAGGATGCAGAAGG	TGCTGGAAGGTGGACAGTGAGG

Figure S1

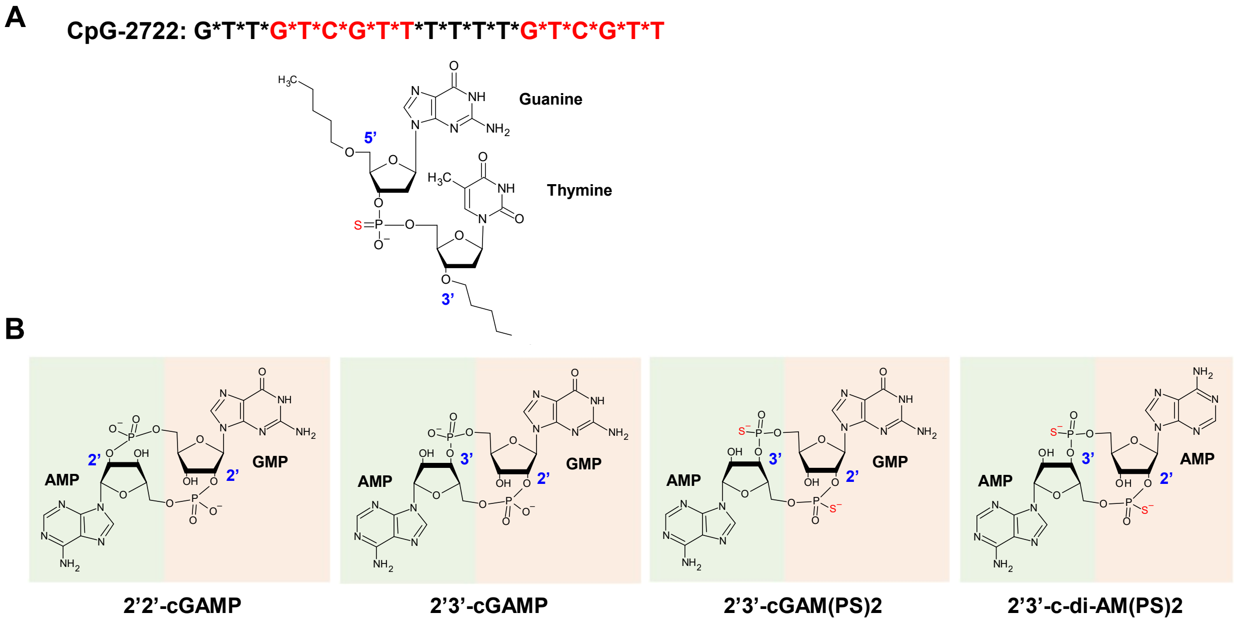

Figure S1. Structural features of TLR9 and STING agonists used in this study. (**A**) CpG-2722 is a TLR9 agonist with a phosphothioated backbone containing 19 nucleotides and two copies of a GTCGTT hexamer motif. Asterisks stand for phosphorothioate bonds. The hexamer motifs are shown in red font. (**B**) Cyclic-dinucleotides (CDNs) for the activation of STING. cGAMP molecules are hybrid CDNs comprising a guanosine monophosphate (GMP) and an adenosine monophosphate (AMP), whereas c-di-AMPs are homodimers of AMP. These CDNs are cyclic by 2'-5'-phosphodiester bonds from the 2'2' or 2'3' positions of the two nucleotides as shown. In contrast with the 2'2'-cGAMP and 2'3'-cGAMP, the 2'3'-cGAM(PS)2 and 2'3'-c-di-AM(PS)2 contain two phosphorothioate modifications as shown in red font.

Figure S2

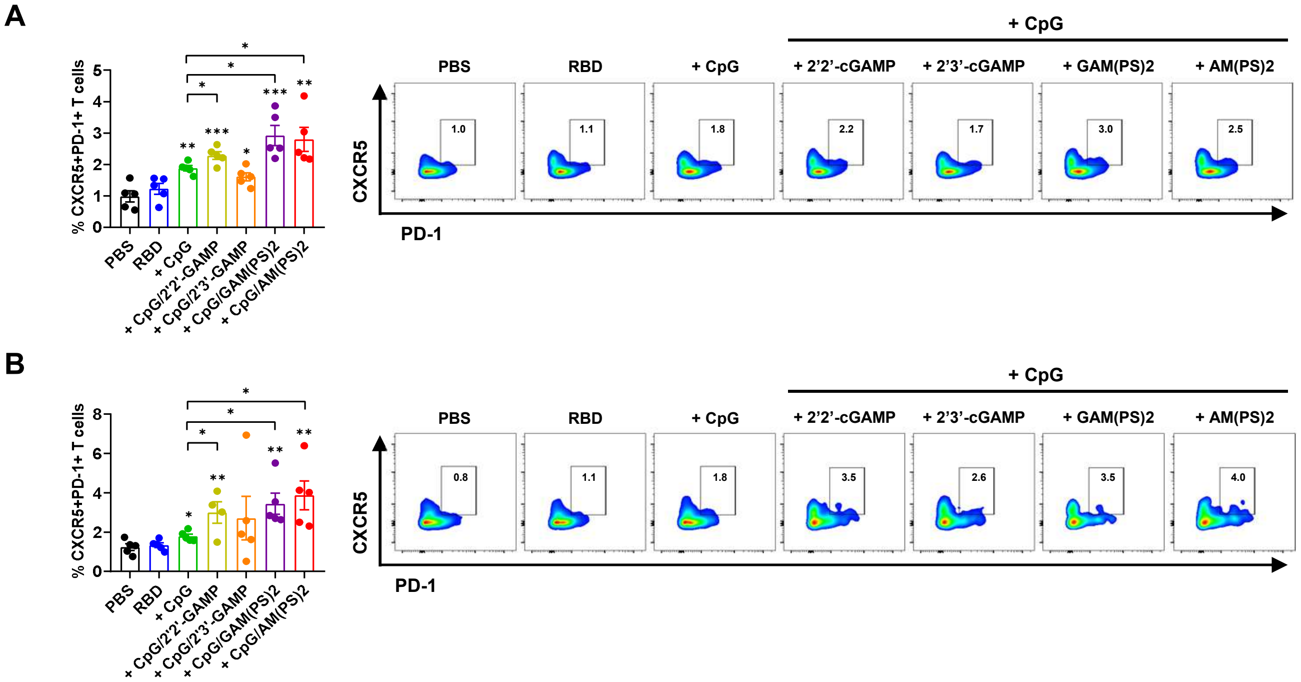

Figure S2. The combination of CpG-2722 and STING ligands elicited robust Tfh cell responses. In the experiments in Figure 1, the mice were euthanized 10 days after the final vaccination (day 30) to collect draining lymph nodes and spleens. The numbers of Tfh cells ($CD4^{+}Bcl\text{-}6^{+}CXCR5^{+}PD\text{-}1^{+}$) from lymph nodes (**A**) and spleens (**B**) were measured by flow cytometry. Data are the mean ± SEM (n = 5/group). *$P < 0.05$, **$P < 0.01$, ***$P < 0.001$, compared with mice treated with PBS vehicle control or between the different groups as indicated. CpG and AM(PS)2 stand for CpG-2722 and 2'3'-c-di-AM(PS)2. + stands for plus RBD protein antigen.

Figure S3

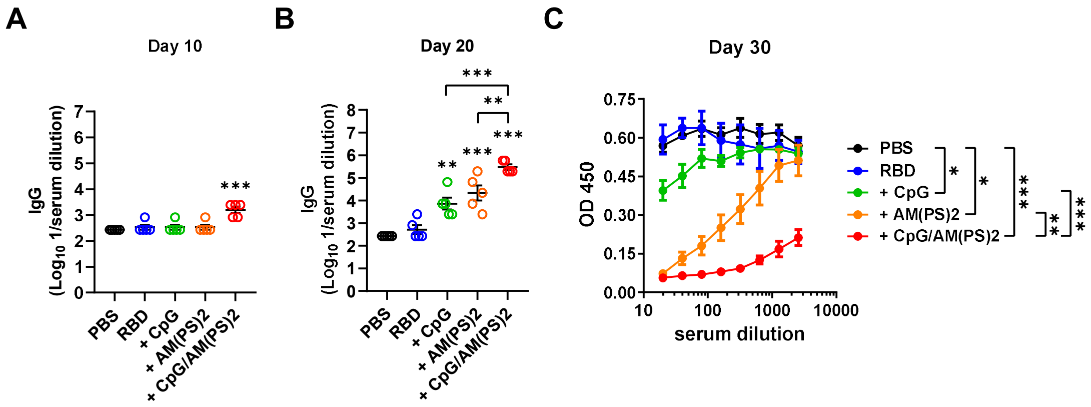

Figure S3. Cooperative adjuvant effect of CpG-2722 and c-di-AM(PS)2 on inducing a humoral response to the RBD protein vaccine. BALB/c mice were immunized intramuscularly on days 0, 11, and 21 with 10 mg of the RBD protein vaccine adjuvanted with 10 μg CpG-2722 and 5 μg c-di-AM(PS)2 alone or in combination. Serum samples were collected on days 10, 20, and 30. These samples collected on day 10 (**A**) and day 20 (**B**) were analyzed for anti-S protein IgG levels by ELISA. (**C**) Serum samples collected on day 30 were subjected to the hACE2-RBD competition assay. Data are the mean ± SEM (n = 5/group). *$P < 0.05$, **$P < 0.01$, and ***$P < 0.001$ compared with mice treated with PBS vehicle control or between the different groups as indicated. CpG and AM(PS)2 stand for CpG-2722 and 2'3'-c-di-AM(PS)2. + stands for plus RBD protein antigen.
